# Supplementary material for: A Likelihood Approach for Real-Time Calibration of Stochastic Compartmental Epidemic Models
Source: PLoS Comput Biol. 2017 Jan 17;13(1):e1005257. doi: 10.1371/journal.pcbi.1005257 (PMC5240920; doi:10.1371/journal.pcbi.1005257)
Supplement: S1 File — (TAR.GZ) [file pcbi.1005257.s014.tar.gz › HSPH_Online-SI-Revision/IPoi09/n1-severe/n1-severe_BENCH_table-4.pdf]

1	R0	{1.51081, 13.7078}	{1.50432, 6.69889}	{{1.42659, 5.32185}, {1.62191, 8.77207}}	{5.35658, 51.1308}	inf	45.	39.375	{8., 68.}	51.5	0.315552	newly	25.	35.65	{7., 67.}	80.2	0.309212	Reff	1.25397	0.846416	{0.613588, 1.12026}	33.2717	ar	656.	586.025	{504., 696.}	13.3346
2	R0	{1.73182, 11.3873}	{1.36539, 5.8999}	{{1.25656, 3.98513}, {1.42446, 7.81552}}	{21.3426, 48.7711}	inf	37.	22.1481	{0., 61.}	68.969	0.843222	newly	33.	22.4815	{0., 55.}	61.9529	0.739354	Reff	1.5621	0.918196	{0.671515, 1.30625}	41.2205	ar	653.	415.185	{59., 580.}	37.2129
3	R0	{1.50449, 10.3474}	{1.44966, 6.11112}	{{1.3618, 4.94504}, {1.51295, 8.4085}}	{5.94929, 42.7749}	inf	32.	37.1143	{12., 67.}	48.3036	0.207628	newly	23.	38.2286	{15., 64.}	79.6273	0.248704	Reff	1.30891	1.03044	{0.707977, 1.34303}	24.8228	ar	651.	532.629	{454., 617.}	19.8157
4	R0	{1.63493, 9.1955}	{2.19495, 16.0187}	{{1.81391, 11.7144}, {2.48947, 19.5287}}	{34.5437, 74.5939}	inf	32.	50.4	{8., 85.}	88.8542	0.348365	newly	23.	26.3333	{4., 45.}	60.7246	0.318881	Reff	1.51231	2.02397	{1.64138, 2.3292}	35.3449	ar	663.	823.333	{735., 908.}	28.2051
5	R0	{1.53822, 8.05553}	{2.35998, 15.7311}	{{1.96373, 11.2959}, {2.78218, 19.8588}}	{53.4229, 95.2835}	inf	44.	108.27	{24., 205.}	162.879	0.399108	newly	31.	55.4603	{14., 99.}	98.3615	0.303879	Reff	1.33671	1.85052	{1.29321, 2.325}	41.0622	ar	621.	868.127	{813., 925.}	39.795
6	R0	{1.69374, 17.0793}	{1.66428, 15.2652}	{{1.514, 12.483}, {1.85782, 19.0269}}	{6.67151, 15.9811}	inf	45.	41.7292	{7., 94.}	53.9352	0.320867	newly	15.	20.3125	{5., 39.}	71.8056	0.288724	Reff	1.54638	1.5081	{1.24061, 1.76356}	9.72702	ar	662.	671.875	{553., 750.}	10.2782
7	R0	{1.42179, 11.8031}	{2.16039, 16.74}	{{1.94617, 13.1357}, {2.30371, 19.4475}}	{51.9488, 41.8405}	inf	53.	154.183	{65., 222.}	198.082	0.469525	newly	34.	65.05	{35., 90.}	100.147	0.311641	Reff	1.16871	1.17563	{0.727598, 1.71981}	26.1008	ar	517.	830.3	{773., 869.}	60.5996
8	R0	{1.46683, 6.95875}	{2.19212, 13.7067}	{{1.83169, 9.75343}, {2.57305, 17.5902}}	{49.5307, 97.7153}	inf	33.	72.2333	{16., 132.}	137.273	0.354163	newly	39.	45.9	{10., 81.}	56.3248	0.260159	Reff	1.32308	1.8558	{1.39575, 2.33338}	41.0291	ar	653.	822.05	{727., 910.}	26.4497
9	R0	{1.34525, 11.505}	{1.84832, 14.9341}	{{1.63746, 10.8293}, {2.12588, 19.2116}}	{37.3961, 32.8653}	inf	25.	89.9804	{14., 145.}	274.353	0.541391	newly	24.	42.9412	{11., 66.}	100.98	0.328293	Reff	1.20669	1.42532	{1.04243, 1.73424}	24.1729	ar	532.	747.314	{644., 834.}	42.5733
10	R0	{1.77958, 14.372}	{2.25857, 17.898}	{{2.08951, 15.9744}, {2.42205, 19.5287}}	{26.9158, 24.8111}	inf	100.	131.397	{49., 209.}	53.1897	0.206712	newly	47.	57.3621	{25., 89.}	45.9648	0.192577	Reff	1.3685	1.58262	{1.12878, 2.0109}	26.5305	ar	671.	853.914	{816., 886.}	27.2599
11	R0	{1.60334, 19.9548}	{1.82051, 15.8779}	{{1.67042, 12.378}, {1.99753, 19.3194}}	{13.783, 20.4307}	inf	56.	91.5208	{43., 140.}	78.3854	0.257474	newly	27.	41.4375	{20., 62.}	68.4414	0.237298	Reff	1.33398	1.24225	{0.839762, 1.741}	20.3333	ar	617.	729.688	{629., 812.}	18.6352
12	R0	{1.68623, 7.58585}	{2.57939, 14.6357}	{{2.14747, 10.7247}, {2.96797, 18.1089}}	{52.9679, 93.0302}	inf	50.	94.6032	{24., 184.}	113.27	0.337109	newly	56.	60.3175	{18., 107.}	51.0204	0.260717	Reff	1.4822	2.0992	{1.60623, 2.59959}	41.6276	ar	640.	884.444	{824., 937.}	38.1944
13	R0	{1.82689, 18.0022}	{2.03585, 17.1091}	{{1.82711, 13.7237}, {2.26107, 19.5128}}	{12.0075, 10.8893}	inf	85.	112.107	{44., 171.}	53.7395	0.226953	newly	30.	48.2321	{23., 70.}	74.8214	0.263264	Reff	1.51084	1.47756	{1.12375, 1.86525}	13.5626	ar	673.	790.036	{722., 866.}	20.7865
14	R0	{1.75911, 9.16883}	{2.34216, 14.2892}	{{1.88056, 9.75343}, {2.81891, 18.4275}}	{33.1444, 55.9336}	inf	22.	55.9474	{15., 107.}	171.372	0.419688	newly	24.	34.386	{12., 65.}	73.538	0.315777	Reff	1.63773	2.1189	{1.66089, 2.57231}	30.3565	ar	661.	845.158	{743., 932.}	31.3427
15	R0	{1.46168, 11.3859}	{2.1112, 15.3111}	{{1.83592, 11.3627}, {2.42205, 19.255}}	{44.4365, 35.4828}	inf	59.	141.709	{68., 215.}	143.883	0.368321	newly	40.	60.6545	{37., 82.}	58.4545	0.19616	Reff	1.10795	0.965097	{0.541512, 1.54661}	30.6472	ar	556.	815.418	{757., 892.}	46.6579
16	R0	{1.53577, 11.7581}	{2.31342, 14.0585}	{{1.85533, 9.28761}, {2.8568, 18.4275}}	{50.8679, 29.1196}	inf	48.	82.3667	{18., 148.}	104.028	0.406023	newly	31.	51.4833	{9., 97.}	97.043	0.387572	Reff	1.38834	1.92662	{1.39521, 2.44518}	39.8713	ar	625.	825.5	{737., 930.}	38.704
17	R0	{1.70698, 9.72924}	{2.0619, 9.08535}	{{1.5451, 4.64686}, {2.66419, 14.2056}}	{25.2612, 31.9552}	inf	51.	89.6271	{28., 161.}	94.3503	0.284624	newly	41.	79.6271	{24., 131.}	109.012	0.316021	Reff	1.46288	1.65979	{0.923014, 2.33383}	29.9225	ar	675.	805.085	{644., 923.}	22.0188
18	R0	{1.5973, 14.931}	{2.10511, 12.9657}	{{1.58886, 7.29751}, {2.56757, 17.5057}}	{33.7968, 22.9254}	inf	33.	49.0952	{9., 90.}	90.7167	0.400065	newly	22.	31.6825	{4., 63.}	86.2915	0.393767	Reff	1.48229	1.97712	{1.41813, 2.4398}	36.3188	ar	632.	798.556	{715., 907.}	33.6423
19	R0	{1.87468, 17.004}	{1.98714, 15.684}	{{1.72065, 11.7288}, {2.21213, 17.7758}}	{8.91763, 12.3745}	inf	109.	129.875	{64., 184.}	40.9404	0.186215	newly	48.	57.5536	{36., 80.}	36.2723	0.159555	Reff	1.37227	1.12102	{0.746183, 1.62289}	26.1349	ar	692.	790.357	{694., 848.}	14.8689
20	R0	{1.84715, 12.531}	{2.23978, 18.8522}	{{2.19504, 17.5123}, {2.30371, 19.6625}}	{21.256, 50.4447}	inf	87.	160.194	{79., 225.}	93.7039	0.288074	newly	54.	60.2836	{37., 84.}	31.3709	0.143864	Reff	1.37797	1.285	{0.754759, 1.8421}	25.5524	ar	661.	848.045	{815., 880.}	28.2972
21	R0	{1.75938, 13.5959}	{1.59916, 5.40224}	{{1.42949, 4.00571}, {1.89254, 8.41615}}	{16.3648, 61.2457}	inf	51.	64.3913	{13., 168.}	71.7818	0.337082	newly	26.	64.0652	{19., 114.}	161.288	0.460786	Reff	1.52538	0.994934	{0.610315, 1.52906}	37.7425	ar	620.	620.304	{470., 858.}	20.6241
22	R0	{1.63456, 10.7469}	{2.11589, 18.0181}	{{1.93882, 15.6958}, {2.26107, 19.9579}}	{29.4469, 67.659}	inf	101.	121.967	{44., 184.}	48.0858	0.220728	newly	65.	49.8833	{16., 73.}	30.4872	0.214151	Reff	1.27823	1.50375	{1.10824, 1.95583}	23.6918	ar	654.	819.383	{774., 862.}	25.288
23	R0	{1.62702, 19.1392}	{1.50558, 5.9333}	{{1.454, 5.4396}, {1.58886, 7.29751}}	{7.76168, 68.9992}	inf	73.	43.7188	{15., 97.}	50.8134	0.368779	newly	31.	41.2813	{15., 85.}	69.0524	0.256766	Reff	1.28209	0.837206	{0.614228, 1.14716}	34.9678	ar	600.	595.625	{510., 673.}	9.5625
24	R0	{1.70466, 12.1202}	{1.65208, 7.75908}	{{1.58886, 7.28757}, {1.70777, 8.49961}}	{3.23717, 35.9822}	inf	59.	31.5952	{5., 63.}	57.9903	0.498408	newly	37.	24.9524	{4., 54.}	55.5985	0.420807	Reff	1.25293	0.690991	{0.521721, 0.889239}	45.6164	ar	613.	649.833	{571., 710.}	8.96838
25	R0	{1.61735, 11.3169}	{1.97786, 19.3874}	{{1.93087, 18.8819}, {1.99771, 19.6444}}	{22.2899, 71.3138}	inf	88.	111.	{45., 162.}	47.9302	0.187886	newly	70.	42.2857	{17., 62.}	39.5918	0.254971	Reff	1.2583	1.33585	{0.905578, 1.74358}	20.8793	ar	681.	789.143	{749., 820.}	15.88
26	R0	{1.56977, 12.3035}	{2.41359, 15.9736}	{{1.96658, 11.2959}, {2.79269, 19.898}}	{53.7542, 33.1494}	inf	74.	177.265	{61., 267.}	148.887	0.383468	newly	43.	78.4706	{44., 104.}	90.1505	0.280209	Reff	1.30605	1.35832	{0.789832, 2.03904}	29.2684	ar	594.	864.721	{780., 932.}	45.5759
27	R0	{1.46317, 5.8298}	{1.46832, 5.83843}	{{1.38889, 4.45823}, {1.58886, 7.29751}}	{3.87049, 15.4414}	inf	28.	39.3824	{12., 62.}	65.4412	0.232384	newly	37.	42.9706	{20., 73.}	45.2305	0.191342	Reff	1.28027	0.911636	{0.68338, 1.22034}	29.7583	ar	586.	544.088	{450., 649.}	12.2917
28	R0	{1.70002, 14.9806}	{2.48401, 16.2658}	{{2.11132, 12.2645}, {2.85956, 19.898}}	{46.1167, 17.1497}	inf	49.	85.6944	{11., 186.}	116.27	0.427177	newly	27.	43.8889	{6., 91.}	104.63	0.422067	Reff	1.54702	2.12048	{1.68, 2.6063}	37.6469	ar	652.	873.264	{834., 930.}	36.6841
29	R0	{1.52729, 16.1014}	{2.16225, 18.3257}	{{1.95665, 15.8893}, {2.26107, 19.5128}}	{41.5744, 15.0185}	inf	86.	156.05	{101., 203.}	83.9341	0.254	newly	43.	57.8167	{36., 73.}	40.2713	0.142629	Reff	1.16379	0.909318	{0.585922, 1.35028}	31.3582	ar	592.	830.45	{765., 880.}	40.2787
30	R0	{1.72795, 13.5635}	{2.24291, 16.4544}	{{2.01902, 13.4708}, {2.46533, 19.391}}	{29.8015, 22.4538}	inf	101.	169.492	{92., 236.}	75.3124	0.243235	newly	52.	72.0164	{44., 91.}	48.4552	0.175821	Reff	1.1992	1.03702	{0.558746, 1.62733}	30.7019	ar	668.	848.607	{800., 894.}	27.0369
31	R0	{1.51591, 18.1544}	{1.94369, 13.8586}	{{1.7434, 10.896}, {2.23964, 18.4504}}	{28.2195, 25.1468}	inf	41.	116.158	{45., 195.}	191.528	0.476496	newly	13.	53.1754	{28., 74.}	315.25	0.630347	Reff	1.27185	1.02277	{0.651383, 1.56648}	29.7069	ar	541.	761.912	{662., 865.}	44.2196
32	R0	{1.4282, 10.3534}	{2.12947, 17.1012}	{{1.82711, 12.8742}, {2.30371, 19.5287}}	{49.1018, 65.4802}	inf	21.	67.5738	{11., 125.}	243.169	0.514253	newly	8.	33.2131	{5., 64.}	332.787	0.603403	Reff	1.31109	1.8355	{1.36625, 2.20228}	42.0959	ar	521.	811.951	{750., 873.}	58.8905
33	R0	{1.56693, 11.3972}	{2.56831, 14.8628}	{{1.93539, 9.63533}, {2.93488, 18.4042}}	{63.907, 36.0299}	inf	51.	77.3788	{15., 150.}	90.9982	0.329182	newly	36.	46.3636	{11., 82.}	70.202	0.316397	Reff	1.38203	2.20964	{1.68362, 2.62065}	60.3087	ar	695.	890.409	{786., 936.}	28.1164
34	R0	{1.50675, 19.8823}	{1.81238, 10.7284}	{{1.75469, 10.0119}, {1.88025, 11.3627}}	{20.284, 46.0403}	inf	72.	69.4894	{24., 112.}	39.2435	0.205263	newly	29.	40.5957	{14., 68.}	60.675	0.220786	Reff	1.0502	0.712291	{0.490024, 1.00058}	36.1257	ar	587.	730.809	{663., 785.}	24.4989
35	R0	{1.72889, 7.76068}	{2.04803, 10.4699}	{{1.40116, 4.45823}, {2.85582, 17.2484}}	{25.7304, 52.2098}	inf	36.	70.1053	{9., 164.}	125.439	0.388052	newly	45.	53.4035	{8., 103.}	66.2378	0.350949	Reff	1.55946	1.79816	{1.13333, 2.54366}	27.9687	ar	650.	781.123	{581., 930.}	27.0823
36	R0	{1.62644, 4.12303}	{1.78494, 4.26734}	{{1.71582, 3.97874}, {1.86981, 4.61852}}	{9.79375, 9.46531}	inf	30.	89.6	{47., 135.}	203.407	0.502104	newly	61.	130.644	{70., 184.}	120.073	0.376829	Reff	1.31091	0.960342	{0.667178, 1.41448}	31.4997	ar	652.	709.089	{662., 807.}	13.7457
37	R0	{1.70878, 15.8072}	{2.0711, 18.1158}	{{1.93288, 15.3647}, {2.1968, 19.9579}}	{21.2034, 15.5774}	inf	96.	140.226	{88., 187.}	56.4465	0.231686	newly	49.	52.283	{37., 68.}	23.7197	0.130499	Reff	1.21665	1.00993	{0.641716, 1.46231}	29.0616	ar	674.	802.811	{772., 857.}	22.6779
38	R0	{1.58967, 6.80568}	{2.12177, 11.7326}	{{1.68297, 7.5059}, {2.73737, 17.2462}}	{33.963, 75.2478}	inf	44.	91.6545	{25., 178.}	130.124	0.385066	newly	51.	64.2727	{15., 108.}	61.533	0.316776	Reff	1.3846	1.70064	{1.07342, 2.36212}	30.4236	ar	610.	805.745	{655., 912.}	32.9478
39	R0	{1.344, 8.19498}	{2.08822, 17.0323}	{{1.79436, 13.0803}, {2.27287, 19.5128}}	{55.3735, 107.838}	inf	37.	109.317	{28., 178.}	210.811	0.478488	newly	32.	48.1587	{12., 73.}	80.1587	0.309592	Reff	1.1545	1.55299	{1.14111, 1.9342}	38.0802	ar	551.	803.968	{722., 873.}	48.8722
40	R0	{1.61509, 7.90825}	{1.60773, 4.11901}	{{1.4804, 3.12918}, {1.81768, 5.68021}}	{6.47581, 48.3143}	inf	38.	73.4468	{30., 123.}	102.912	0.283034	newly	48.	115.277	{70., 165.}	144.504	0.373275	Reff	1.39544	1.09048	{0.723916, 1.43984}	25.7756	ar	581.	664.255	{553., 795.}	17.479
41	R0	{1.60017, 5.90605}	{2.36315, 15.893}	{{2.08003, 12.4507}, {2.7811, 19.8588}}	{47.681, 169.096}	inf	46.	96.4242	{25., 184.}	131.159	0.361298	newly	81.	47.9394	{12., 87.}	44.1452	0.362182	Reff	1.38735	1.93163	{1.52407, 2.42609}	40.935	ar	661.	867.394	{806., 927.}	31.2245
42	R0	{1.64436, 15.1337}	{1.81123, 9.1433}	{{1.47651, 5.4396}, {2.29055, 14.0824}}	{15.1953, 42.3319}	inf	52.	93.6889	{27., 155.}	103.162	0.317197	newly	26.	66.3333	{26., 110.}	162.137	0.393169	Reff	1.38455	1.28476	{0.752212, 1.90029}	26.2084	ar	640.	722.222	{530., 900.}	20.3542
43	R0	{1.48917, 11.917}	{2.02079, 19.3089}	{{1.98971, 19.0665}, {2.11476, 19.6444}}	{35.6993, 62.0283}	inf	67.	117.263	{37., 174.}	89.7355	0.281041	newly	42.	44.4211	{19., 63.}	33.584	0.162229	Reff	1.16602	1.2793	{0.817063, 1.80593}	25.5713	ar	551.	801.053	{759., 848.}	45.3816
44	R0	{1.65577, 7.89317}	{1.72076, 8.99937}	{{1.31748, 4.25564}, {2.22867, 15.4278}}	{19.2917, 48.6636}	inf	24.	71.3265	{19., 183.}	208.759	0.409959	newly	30.	52.8776	{15., 101.}	95.034	0.285679	Reff	1.52331	1.36564	{0.770795, 2.01971}	25.5892	ar	576.	671.449	{445., 878.}	30.7929
45	R0	{1.6909, 12.3275}	{2.0187, 12.633}	{{1.81647, 10.0119}, {2.22641, 14.8348}}	{19.3859, 13.9151}	inf	89.	120.382	{53., 178.}	56.9561	0.240343	newly	56.	67.5455	{25., 95.}	45.1623	0.215677	Reff	1.29354	1.32825	{0.821268, 1.82008}	23.8291	ar	675.	799.745	{725., 860.}	18.5778
46	R0	{1.62351, 16.0698}	{1.7242, 13.2297}	{{1.52414, 9.52864}, {1.99485, 17.8178}}	{10.2103, 22.5726}	inf	41.	80.8302	{19., 146.}	120.709	0.399974	newly	19.	41.3962	{7., 67.}	141.509	0.443039	Reff	1.43681	1.26538	{0.878212, 1.71626}	20.8686	ar	591.	691.736	{606., 803.}	21.7572
47	R0	{1.52372, 4.38599}	{2.30158, 12.311}	{{1.50982, 5.32185}, {2.85582, 16.8013}}	{52.6286, 181.709}	inf	11.	45.629	{5., 99.}	334.164	0.620626	newly	26.	35.2419	{3., 75.}	85.2978	0.41876	Reff	1.44449	2.16777	{1.46658, 2.73613}	53.2657	ar	618.	814.355	{661., 935.}	42.3478
48	R0	{1.44306, 11.788}	{2.25904, 16.1794}	{{2.01902, 13.0262}, {2.49423, 19.3027}}	{56.545, 37.7853}	inf	53.	168.81	{94., 235.}	222.162	0.495572	newly	34.	73.7937	{51., 94.}	121.989	0.353168	Reff	1.12126	1.16827	{0.714946, 1.77359}	27.5567	ar	534.	849.	{783., 889.}	58.9888
49	R0	{1.63624, 11.3893}	{1.96243, 10.5584}	{{1.47651, 5.4396}, {2.56757, 16.884}}	{23.7268, 29.9275}	inf	71.	94.6	{27., 154.}	65.5869	0.297535	newly	46.	66.4167	{23., 109.}	68.8768	0.28631	Reff	1.39244	1.50276	{0.823893, 2.1392}	32.0726	ar	643.	763.483	{588., 901.}	25.254
50	R0	{1.46113, 11.1322}	{1.78302, 13.393}	{{1.51295, 8.4085}, {2.00049, 16.7932}}	{22.7565, 29.9771}	inf	34.	58.32	{10., 114.}	104.	0.359522	newly	19.	32.32	{5., 58.}	101.895	0.362393	Reff	1.33547	1.52739	{1.13219, 1.83986}	20.923	ar	501.	699.48	{564., 820.}	47.0739
